# Malaria Transmission at The Zimbabwe–Mozambique Border: An Observational Study of Parasitemia by Travel History and Household Location

**DOI:** 10.4269/ajtmh.23-0466

**Published:** 2024-05-21

**Authors:** Ellen Ferriss, Sungano Mharakurwa, Shungu Munyati, Lovemore Gwanzura, Marisa A. Hast, Lawrence H. Moulton, Amy Wesolowski, William J. Moss

**Affiliations:** ^1^ Department of International Health, Johns Hopkins Bloomberg School of Public Health, Baltimore, Maryland;; ^2^ Africa University, Mutare, Zimbabwe;; ^3^ Biomedical Research and Training Institute, Harare, Zimbabwe;; ^4^ University of Zimbabwe, Harare, Zimbabwe;; ^5^ Department of Epidemiology, Johns Hopkins Bloomberg School of Public Health, Baltimore, Maryland;; ^6^ Department of Biostatistics, Johns Hopkins Bloomberg School of Public Health, Baltimore, Maryland;; ^7^ W. Harry Feinstone Department of Molecular Microbiology and Immunology, Johns Hopkins Bloomberg School of Public Health, Baltimore, Maryland

## Abstract

Cross-border human population movement contributes to malaria transmission in border regions, impeding national elimination. However, its impact in low-to-moderate transmission settings is not well characterized. This community-based study in Mutasa District, Zimbabwe, estimated the association of parasite prevalence with self-reported overnight travel to Mozambique and household distance to the border from 2012–2020. A fully adjusted Poisson regression model with robust variance estimation was fit using active surveillance data. The population attributable fraction of parasite prevalence from overnight travel was also estimated. The relative risk of testing positive for malaria by rapid diagnostic test declined 14% (prevalence ratio [PR] = 0.86, 95% CI = 0.81–0.92) per kilometer from the border up to 12 km away. Travel to Mozambique was associated with a 157% increased risk (PR = 2.57, 95% CI = 1.38–4.78), although only 5.8% of cases were attributable to overnight travel (95% CI = −1.1% to 12.7%), reflecting infrequent overnight trips (1.3% of visits). This study suggests that transmission in eastern Zimbabwe is driven by increasingly conducive social or environmental conditions approaching the border and low levels of importation from overnight travel. Although day trips to Mozambique during peak biting hours were not assessed, the contribution of such trips to ongoing transmission may be significant. Future malaria control efforts should prioritize high coverage of existing interventions and continued support for community health workers and health facilities at the border, which provide free case management.

## INTRODUCTION

In 2021, Zimbabwe was estimated to have 342,543 confirmed malaria cases and 876 malaria deaths.^1^ Despite three-quarters of the population living in malaria-free or low-transmission settings, malaria continues to pose a public health challenge along the country’s northern and eastern borders with Mozambique, where transmission is higher.^2^ In 2016, 82% of cases occurred in 3 such border provinces, among them Manicaland Province, which reported 39% of cases and 31% of malaria deaths nationally.^3^

Cross-border human population movement between Zimbabwe and Mozambique is believed to contribute to elevated transmission at the border. In Mutasa District, Manicaland Province, individuals regularly travel to or from Mozambique to visit friends and family, shop, engage in work, seek healthcare, and attend school (L. Siems, unpublished manuscript, 2016).^4^ Previous surveys have estimated roughly 15% to 25% of the population travels to Mozambique overnight monthly, and only 20% to 40% take measures to prevent malaria (e.g., use bed nets, repellents, and covering clothing).^4^^,^^5^

Zimbabwe’s National Malaria Control Program (NMCP) has identified strengthening border malaria control as an area for program improvement.^3^ The NMCP has prioritized case management for mobile and migrant populations and has developed targeted social and behavior change communications, in addition to preventive and curative service strategies.^3^ Zimbabwe is additionally party to the Elimination 8 Regional Initiative (E8) to advance malaria elimination in southern Africa, which has prioritized the reduction of cross-border transmission as a core strategic objective.^6^ The E8 has established surveillance teams and seven mobile malaria posts for outpatient care along the Zimbabwe–Mozambique border.^5^ Apart from these efforts, formal cooperation on malaria control between Zimbabwe and Mozambique has been limited.^4^

**Figure 1.**
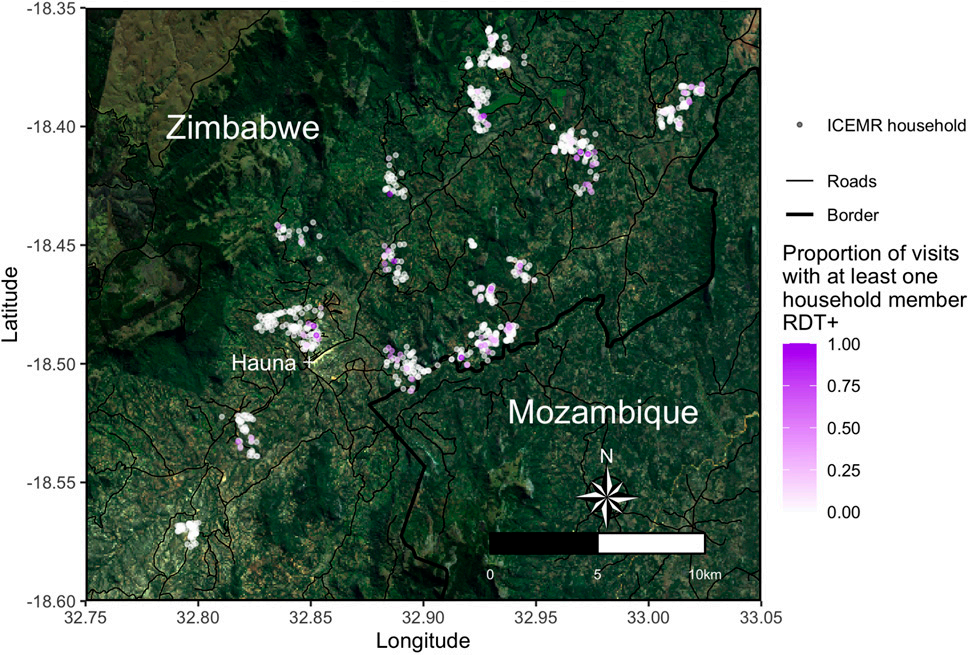
The proportion of household visits during which at least one household member was positive for malaria by rapid diagnostic test from 2012 through 2020 at the International Centers of Excellence for Malaria Research (ICEMR) study site in Mutasa District, Zimbabwe. RDT = rapid diagnostic test.

Despite the potential risk posed by cross-border malaria, few studies have sought to characterize it in Zimbabwe and other parts of southern Africa with low endemicity that are not yet in the elimination phase. This study models the associations of overnight travel to Mozambique and household proximity to the border with *Plasmodium falciparum* parasite prevalence at the Southern and Central Africa International Centers of Excellence for Malaria Research (ICEMR) study site in Mutasa District, Zimbabwe (Figure 1).

## METHODS AND MATERIALS

### Study area.

Mutasa District is located in Manicaland Province along Zimbabwe’s eastern border with Mozambique. The district encompasses Honde Valley in the northeast and the Eastern Highlands mountain range in the west, corresponding to elevations ranging from 600 m to more than 1,800 m above sea level (asl). Honde Valley, which spans the border into Manica Province, Mozambique, has a humid subtropical climate with average daily temperatures ranging from 16.3°C in July to 24.5°C in November.^7^^,^^8^ The rainy season occurs from November through April and the dry season from May through October. The population practices subsistence or smallholder commodity farming and plantation agriculture on tea, coffee, and banana estates.^9^

Malaria in Mutasa District is primarily caused by the parasite *P. falciparum* and occurs seasonally, with outbreaks occurring during and immediately after rainy season onset.^10^ Malaria control consists of case management, intermittent preventive treatment of malaria during pregnancy, and vector control with indoor residual spraying (IRS) and long-lasting insecticidal nets (LLINs). Case management is conducted by community health workers and at health facilities, with treatment provided free-of-charge to both nationals and nonnationals.^4^ As of 2014, 92% of the district’s population was protected by IRS, and from 2012 to 2017, 31.8% of individuals surveyed at the ICEMR study site reported sleeping under a bed net.^11^^,^^12^ Before the 2014 IRS campaign, pyrethroids were used for spraying. However, with the development of insecticide resistance, the NMCP transitioned to the organophosphate pirimiphos-methyl (Actellic^®^ 300CS; Syngenta AG, Basel, Switzerland) in 2014 and DDT in November 2018, both of which are effective against the predominant indoor vector, *Anopheles funestus*.^13^^,^^14^

### Data collection and preparation.

Community-based active surveillance was conducted by the Southern and Central Africa ICEMR, led by the Biomedical Research and Training Institute and Africa University, from November 2012 through March 2020, as described elsewhere.^8^ Household enumeration, selection, and survey administration were conducted for two study arms, cross-sectional and longitudinal, in alternating months. Households were enumerated using high-resolution satellite imagery (Digital Global Services, Inc., Denver, CO), selected through random sampling of 1 km × 1 km grid cells with oversampling in low-population-density cells, and assigned a study arm. Recruitment into both arms was ongoing through September 2017 and limited to the cross-sectional arm thereafter. All present, consenting individuals aged 3 months or older were enrolled from sampled households. Demographic, baseline socioeconomic, malaria prevention, and travel information were collected from participants via survey at household visits. A *P. falciparum* histidine-rich protein 2–based rapid diagnostic test (RDT; SD Bioline; Standard Diagnostics, Yongin, South Korea) was administered at each visit, and test-positive individuals were offered artemether/lumefantrine, or quinine and clindamycin if in the first trimester of pregnancy, in accordance with national guidelines. Participant responses, RDT results, and household coordinates were recorded on Android tablets using Open Data Kit (www.opendatakit.org).

GPS logger data were obtained for a subset of individuals who participated in the ICEMR’s study on population mobility from June 2016 through August 2017, as described elsewhere.^15^ In brief, participants aged 13 years and older were enrolled for ∼1 month, during which time they carried a GPS logger that recorded their location at 5-min intervals while in motion. Logger study participants were considered to have traveled overnight if their final recorded geocoordinate of 1 day and their first 10 geocoordinates of the following day were in Mozambique.

Participant age, sex, LLIN use, IRS receipt in the past 6 months, head of household educational attainment, asset ownership, floor type, elevation, and overnight travel to Mozambique in the past 4 weeks were obtained from the ICEMR’s surveillance data. IRS period was modeled as a binary indicator, defined as pyrethroid (October 2012–October 2014) or post-pyrethroid period (November 2014–March 2020), to capture the decline in parasite prevalence following the transition to primiphos-methyl and subsequently DDT.^16^

Environmental data were obtained from publicly available sources and processed in R version 4.1.0 (R Core Team, Vienna, Austria) and QGIS version 3.10.7 (QGIS Development Team, Gossau, Switzerland). Household distance to the border was calculated in R using the *sf* package with Humanitarian Data Exchange’s Zimbabwe administrative level 0 shapefile.^17^ Distance to the nearest first-, second-, third-, and fourth-order streams were calculated in QGIS using the GRASS v.distance algorithm from a stream network classified according to the Strahler system, which was generated from a 90-m resolution digital elevation model (DEM) from NASA’s Shuttle Radar Topography Mission.^18^ Household slope and aspect were calculated in QGIS using the DEM. Landsat 8 Operational Land Imager (OLI) and Thermal Infrared (TIRS) Collection 2 Level 2 satellite imageries were obtained from September 2013 through April 2020 to calculate seasonal normalized difference vegetation index (NDVI). A satellite image was obtained for April and October each year, corresponding to the end of the rainy and dry seasons, respectively. Where cloud cover precluded using April or October satellite imagery, data without cloud cover was used from either the previous or following month, as available. Seasonal NDVI was calculated from the near infrared (NIR) and red (RED) spectral bands as NDVI = (NIR –Red) / (NIR + Red), with April NDVI assigned to observations from November through the end of April and October NDVI assigned to observations from May through the end of October. Daily 0.05° × 0.05° resolution rainfall data from Climate Hazards group Infrared Precipitation with Stations (CHIRPS) were obtained using the *chirps* R package, and daily 0.5° × 0.625° latitude and longitude global grid temperature data were obtained from the NASA Langley Research Center POWER Project funded through the NASA Earth Science Directorate Applied Science Program using the *nasapower* R package.^19^^,^^20^ Lagged rainfall and minimum temperature were modeled as continuous seasonal confounders. Optimal lags were identified by iteratively regressing RDT status on rolling 7-day-averaged daily rainfall and minimum temperature, lagged 1 to 90 days, using the *geepack* R package.^21^^–^^23^ Regression models were fit for high and low transmission seasons, defined as December through May and June through November, respectively. The lag times that minimized model quasi-likelihood under the independence model criterion (QIC) were tested in the final model and those that minimized QIC selected.

## STATISTICAL ANALYSES

Changes in parasite prevalence associated with self-reported overnight travel to Mozambique in the previous 4 weeks and per kilometer increase in household distance from the border were estimated. A Poisson regression model with robust variance estimation was fit using generalized estimating equations to account for all within-household correlation using *geepack* with an exchangeable working correlation structure. Participant RDT result was specified as the outcome. The model was adjusted for potential demographic, behavioral, and environmental confounders identified from the literature and preliminary data exploration.^8^^,^^9^^,^^24^

The fraction of malaria cases attributable to overnight travel to Mozambique—that is, the population attributable fraction (PAF)—was estimated using Levin’s formula. Two travel frequency estimates (Pe), survey-based and logger study-based, and the adjusted parasite prevalence ratio (PR) associated with travel from the model were used^25^:

$$\text{PAF}=\frac{Pe\;X\;(PR-1)}{Pe\;X\;(PR-1)+1}.$$

Self-reported travel frequency was calculated as the average participant’s fraction of visits at which they reported overnight travel to Mozambique. GPS logger travel frequency was calculated as the average logger participant’s fraction of 28-day travel periods with recorded overnight travel and used as an estimate for the broader study population. Then, 95% bootstrap CIs were generated by simulating 2,000 new sample populations. For each sample, the final regression model was refit to obtain the PR associated with overnight travel, travel frequency estimates recalculated, and two estimates of the PAF generated.

### Ethical considerations.

Study approval was granted by the Biomedical Research and Training Institute (AP102/11) and Johns Hopkins Bloomberg School of Public Health (IRB# 3467) institutional review boards and the Medical Research Council of Zimbabwe (MRCZ/A/1625). Approval was sought from local community leaders prior to survey administration and separately before GPS logger data collection. Written informed consent was obtained from study participants at least 16 years of age and from caretakers of participants under age 16.

## RESULTS

From October 2012 through March 2020, 3,179 participants from 1,004 households were observed, for a total of 6,840 participant visits (Table 1). One hundred thirty longitudinally surveyed households contributed 4,365 observations, and 874 cross-sectionally surveyed households contributed 2,475 observations. Among participants with complete survey data for this analysis, 179 were tracked for approximately 1 month between June 2016 and August 2017 as part of the logger study.

**Table 1:** The number of participants and households observed from 2012 to 2020, tabulated according to total visits

Total Visits	Participants	Households
1	2,819	879
2	139	91
3	33	4
4	10	0
5	9	2
6	9	1
7	8	0
8	7	0
9	6	0
10	5	0
11	5	0
12	0	1
13	6	0
14	4	0
15	3	0
16	5	0
17	5	0
18	5	0
19	4	0
20	3	0
21	7	0
22	8	1
23	6	0
24	9	0
25	6	0
26	6	0
27	4	0
28	6	1
29	2	0
30+	40	24

**Figure 2.**
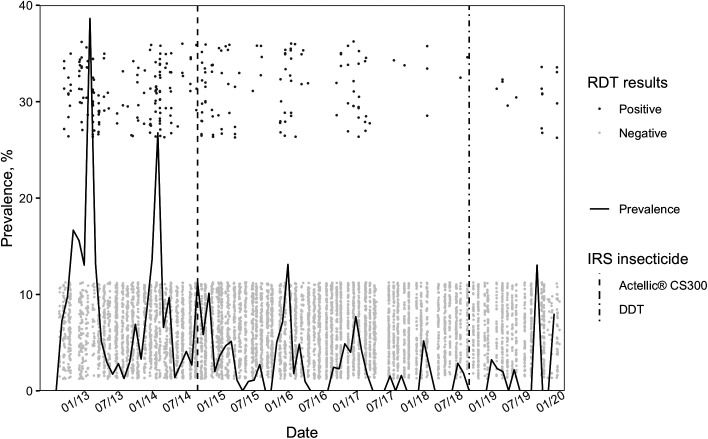
Monthly parasite prevalence by rapid diagnostic test from 2012 through 2020. Indoor residual spraying (IRS) insecticide transitions are shown by vertical lines. Participant test results are jittered, with positive tests above and negative tests below. RDT = rapid diagnostic test.

Seasonal peak malaria prevalence decreased from nearly 40% in April 2013 to a low of 3% in March 2019 (Figure 2). Higher parasite prevalence was recorded from 2012 to 2014 when pyrethroids were used in IRS. Following the transition to Actellic in November 2014, transmission declined and remained low through mid-2019. However, in November 2019, a year after the first application of DDT, parasite prevalence increased to a seasonal high of 11%.

Of the nearly 3,200 participants, only 70 from 45 households reported traveling overnight to Mozambique during the previous 4 weeks for a total of 89 visits. Eleven of 179 logger study participants were recorded traveling overnight to Mozambique, seven of whom were surveyed within 4 weeks of returning. Only 4 of 7 correctly reported recent travel, and of the four individuals who were not surveyed, two did not participate because they were traveling during the scheduled follow-up visit, highlighting the challenge of using home-based surveys to capture travel events.

Half of all households were located within 5 km of the border (Q1–Q3 = 1.7–8.8 kilometers). Household elevation ranged from 600 to more than 1,200 m asl with a median elevation of 760 m asl. Median participant age at first visit was 20 years old (minimum–maximum = <1–99 years old) across the study population and 35 years old (minimum–maximum = 13–88 years old) among logger study participants. Fifty-seven percent of all participants and 55% of logger study participants were female. Twenty-eight percent of households did not own a radio, cell phone, television, refrigerator, or solar panel at the baseline visit, and 16% had floors made of earth, mud, or dung. Individuals reported using an LLIN at 33% of visits, and households reported receiving IRS in the previous 6 months at 38% of visits and 61% of high transmission season visits.

Participant characteristics across study visits according to self-reported overnight travel to Mozambique and household distance to the border are shown in Table 2. Overnight travel was more commonly reported at visits among participants who were older than 20 years and those living closer to the Mozambique border. Participants living within 5 km of the border reported recent overnight travel at 2.1% of visits compared with 0.3% among those living farther away. LLIN use was reported less often the month in which travel occurred during both low and high transmission seasons (22.5% of total visits with reported travel compared with 33.3% of visits with no reported travel). Male participants and individuals from households with lower asset ownership contributed comparatively more observations within 5 km of the border (59.4% versus 52.0% and 42.1% versus 28.5%, respectively). Environmental characteristics—elevation, NDVI, and distance to second-order streams—and malaria control measures, namely, reported LLIN use and IRS receipt in the past 6 months, were comparable in border and inland areas.

**Table 2:** Participant characteristics at each visit according to household distance from the border and self-reported overnight travel to Mozambique in the past 4 weeks

Characteristic	Lives within 5 km of Border		Lives at Least 5 km from Border	
	No Travel *n* = 3,730	Travel *n* = 81	No Travel *n* = 3,021	Travel *n* = 8
Age in years, median (Q1–Q3)	21.0 (7.0–36.0)	27.0 (22.0–37.0)	21.0 (10.0–44.0)	38.0 (26.5–44.2)
Female, *n* (%)	1,515 (40.6%)	32 (39.5%)	1,450 (48.0%)	3 (37.5%)
Head of household educational, *n* (%)				
Primary education	1,520 (40.8%)	34 (42.0%)	1,407 (46.6%)	4 (50.0%)
Secondary education or higher	2,210 (59.2%)	47 (58.0%)	1,614 (53.4%)	4 (50.0%)
Floor type, *n* (%)				
Finished flooring (parquet, tiles, brick, ceramic, concrete, carpet)	3,115 (83.5%)	73 (90.1%)	2,555 (84.6%)	7 (87.5%)
Natural (earth, mud, dung)	615 (16.5%)	8 (9.9%)	466 (15.4%)	1 (12.5%)
No radio, cell phone, television, refrigerator, or solar panel in household, *n* (%)	1,576 (42.3%)	29 (35.8%)	860 (28.5%)	2 (25.0%)
Sleeps under LLIN, N (%)	1,227 (32.9%)	20 (24.7%)	1,027 (34.0%)	0 (0.0%)
House received IRS in past 6 months, N (%)	1,416 (38.0%)	30 (37.0%)	1,172 (38.8%)	1 (12.5%)
IRS period, *n* (%)				
Pyrethroid	1,133 (30.4%)	18 (22.2%)	986 (32.6%)	2 (25.0%)
Post-pyrethroid (pirimiphos-methyl or DDT)	2,597 (69.6%)	63 (77.8%)	2,035 (67.4%)	6 (75.0%)
High transmission season, *n* (%)	1,862 (49.9%)	35 (43.2%)	1,536 (50.8%)	2 (25.0%)
Elevation, m, median (Q1–Q3)	778.0 (724.0–803.1)	753.0 (726.0–803.0)	774.0 (764.3–813.5)	840.0 (772.0–896.5)
NDVI, median (Q1–Q3)	0.2 (0.2–0.3)	0.2 (0.2–0.3)	0.3 (0.2–0.3)	0.2 (0.2–0.3)
Kilometers to second-order stream, median (Q1–Q3)	1.3 (1.1–1.6)	1.2 (0.5–1.6)	1.2 (0.5–1.5)	0.8 (0.6–1.4)

LLIN = long-lasting insecticidal nets; NDVI = normalized difference vegetation index; Q1 = 1st quartile; Q3 = 3rd quartile.

Overnight travel to Mozambique in the past 4 weeks was associated with a 157% higher risk (relative risk [RR] = 2.57, 95% CI = 1.38, 4.78) of testing positive for malaria by RDT, after adjusting for confounders (Table 3). Using self-reported travel history, an estimated 2.6% of malaria cases were attributable to overnight travel to Mozambique (95% bootstrap CI = −0.5 to 5.3%), compared with 5.8% (95% bootstrap CI = −1.1 to 12.7%) using travel frequency collected from GPS loggers. After restricting the analysis to participants living within 5 km of the border, the estimated proportion of cases attributable to overnight travel increased to 5.1% using self-reported travel history and 10.2% using logger data. Living closer to the border was statistically significantly associated with increased risk of being RDT positive after controlling for environmental covariates. The risk of parasitemia decreased 14% (RR = 0.86, 95% CI = 0.81–0.92) per additional kilometer from Mozambique.

**Table 3:** Unadjusted (left) and adjusted (right) associations of prevalent malaria by rapid diagnostic test with self-reported overnight travel to Mozambique in the past 4 weeks, household distance to the border, and potential confounders from Poisson regression models with robust variance estimation

	Unadjusted			Adjusted		
Characteristic	PR	95% CI	*P*-Value	PR	95% CI	*P*-Value
Traveled to Mozambique	2.64	1.37–5.08	0.004	2.57	1.38–4.78	0.003
1 km increase in household distance to border	0.89	0.84–0.93	<0.001	0.86	0.81– 0.92	<0.001
Age, years	0.98	0.97–0.98	<0.001	0.98	0.97–0.99	<0.001
Female	0.75	0.58–0.97	0.029	0.78	0.63–0.98	0.031
Head of household received secondary education or higher	0.82	0.61–1.12	0.217	0.75	0.57–0.99	0.044
Natural (earth, mud, dung) floor	2.79	1.99–3.91	<0.001	1.22	0.88–1.70	0.228
No radio, cell phone, television, refrigerator, or solar panel in household	2.24	1.66–3.03	<0.001	1.61	1.21–2.13	<0.001
Sleeps under LLIN	1.36	0.99–1.87	0.057	1.01	0.76–1.33	0.966
House sprayed in past 6 months	1.86	1.38–2.52	<0.001	1.33	0.97–1.81	0.075
Pyrethroid IRS period, compared with Acetllic^®^ 300CS/DDT	3.74	2.77–5.06	<0.001	4.47	3.29–6.07	<0.001
Elevation, per 100 m	0.55	0.41–0.72	<0.001	0.75	0.57–0.98	0.035
NDVI, 0.1-unit increase	1.66	1.39–1.99	<0.001	1.12	0.91–1.38	0.295
Kilometers from second-order stream	1.74	1.38–2.19	<0.001	1.24	1.01–1.53	0.041
High transmission season	3.08	2.18–4.35	<0.001	1.35	0.80–2.29	0.259
12-day lagged rainfall, 10-mm increase	1.26	1.06–1.50	0.010	5.25	1.96–14.04	<0.001
Interaction: 12-day lagged rainfall, 10-mm increase × high transmission season	–	–	–	0.16	0.06–0.43	<0.001
71-day lagged minimum temperature, °C	1.23	1.17, 1.30	<0.001	1.22	1.14–1.30	<0.001

IRS = indoor residual spraying; LLIN = long-lasting insecticidal nets; NDVI = normalized difference vegetation index; PR = prevalence ratio.

Demographic and environmental confounders included participant age, sex, head of household educational attainment, household asset ownership, distance to surface water, rainfall, and temperature. The risk of testing positive for malaria decreased 2% per year increase in age, and females were 22% less likely to be parasitemic than males. Lower head of household educational attainment and the absence of a radio, cell phone, television, refrigerator, and solar panel in the home were risk factors. Increasing average rainfall and minimum temperature were also associated with increased parasite prevalence approximately 2 weeks and 2 months later, respectively. Living at higher elevation and closer to a second-order stream were negatively associated with risk. Measures of malaria control—that is, self-reported LLIN use and living in a sprayed house in the past 6 months—were not statistically significantly associated with parasite prevalence. However, living in a house that received IRS in the past 6 months was associated with testing positive for malaria, a finding consistent with previous studies and one that may reflect increased baseline risk among households that were targeted for IRS in years where spraying was not universal, higher risk among households that accepted IRS, or residual seasonal confounding.^9^^,^^24^ Finally, participants observed while pyrethroids were used in IRS were estimated to have a 4.5-fold higher parasite prevalence compared with those observed when Actellic^®^ 300CS or DDT was used.

## DISCUSSION

Overnight travel to Mozambique and household proximity to the border were significant risk factors for malaria in this area of eastern Zimbabwe, highlighting the need for additional interventions that protect travelers and the border community. Despite the risk associated with travel, this analysis modeled most malaria cases as locally acquired.

Malaria control programs and studies across southern Africa have documented the impact of cross-border travel on malaria transmission.^26^^–^^31^ In northern Namibia, travel to Zambia or Angola was associated with nearly 11-fold higher odds of malaria in one study, and in a second study, travel to Angola was associated with 44-fold higher odds of malaria in men.^26^^,^^27^ In Eswatini, travel to Mozambique was associated with 4.4-fold higher odds of being seropositive to *P. falciparum* antigens and up to a 57-fold higher odds of being a case.^28^^,^^29^ Additional research in Eswatini found 38% of investigated cases were imported, and distance to a locally or internationally imported case was the second most predictive risk factor for malaria during the high transmission season.^30^ Finally, one study in an endemic border district of Kwazulu-Natal, South Africa, found all cases detected through community-based active surveillance were imported from Mozambique, as were all cases detected at a local border market.^31^ Although these studies highlight the challenge importation poses to malaria control and elimination in very low transmission areas, they stand in contrast to the findings of our study. Among residents of Mutasa District, the elevated risk of testing positive for malaria within 4 weeks of overnight travel to Mozambique was, by comparison, modest. Our analysis suggests importation events may not be a key driver of border malaria in certain low transmission settings, unlike in areas approaching elimination.

The PAF associated with overnight travel to Mozambique represented a small percentage of actively detected malaria cases. However, several limitations of the estimate are noted. Cases were diagnosed using RDT, which is less sensitive than polymerase chain reaction, likely resulting in misclassification of low parasitemia infections as noncases. Further, individuals who traveled within a week of survey administration and contracted malaria would have tested negative, given the absence of antigenemia during the intrinsic incubation period, precluding infection ascertainment in at least one-quarter of traveling participants. In addition, detected cases may have been misclassified as imported or local: RDT-positive individuals who reported overnight travel to Mozambique in the previous 4 weeks were considered imported cases, although some were presumably autochthonous, whereas those who traveled to Mozambique during peak biting hours but did not spend the night or who traveled more than 4 weeks earlier may have been infected in Mozambique but misclassified as a local case. In addition, two limitations of the travel frequency estimates are noted. First, the proportion of individuals who traveled overnight was underestimated via survey due to both misreporting and selection bias, confirmed by discrepancies between GPS logger data and self-reported travel. Second, logger data, although potentially more accurate than self-report, were only available for a subset of participants who were sampled to maximize geographic representation and balance across age and sex categories, thus may not have been representative of the study population. Because travel was more common among adults and the logger study did not include children under 13 years old, travel frequency was likely overestimated. In addition, logger data were only available from 2016 through 2017, during which time self-reported overnight travel was at its highest, thus may not have reflected travel patterns for the duration of the study. Nonetheless, the PAFs estimated using two sources of travel frequency data provide possible ranges.

While the modeled contribution of travel to parasite prevalence was minimal, this study estimated a markedly lower frequency of overnight travel to Mozambique than previous studies in the area, suggesting heterogeneity in importation across the district. Research conducted by the Ministry of Health and Child Care in 2015 estimated 23% of the border population in Manicaland Province traveled overnight to Mozambique monthly.^4^ Similarly, research conducted by the NMCP in Mutasa District in 2018 estimated 16% of adults traveled overnight at least once in the past 3 months, with an average of more than one trip per month and with 89% of such trips reported to Mozambique.^5^ These findings suggest the contribution of travel to locally identified malaria cases is greater in border communities than estimated in our analysis, which assessed a broader geographic area. Indeed, after restricting to participants living within 5 km of the border, the estimated proportion of cases attributable to travel increased from 2.6% to 5.1% using self-reported travel history and from 5.8% to 10.2% using logger data. Malaria control interventions targeting travelers in eastern Zimbabwe should likely be tailored to communities within walking distance of Mozambique.^5^

Living closer to the border was associated with higher parasite prevalence, reflecting ecological variation and possibly unmeasured cross-border human and vector movement. Previous research at the site similarly estimated 14% lower odds of parasitemia per kilometer increase in household distance from Mozambique without adjusting for international travel, suggesting increased risk approaching the border was not driven by importation events.^24^ Other studies in southern Africa have demonstrated graded transmission intensity.^27^^,^^32^ In north-central Namibia, individuals living within 15 km of Angola were estimated to have 186% increased odds of contracting malaria compared with those living farther away.^27^ In Angola, the odds of fever were 55% lower among children living within 5 km of the Namibian border, a risk that increased farther inland.^32^ In our analysis, elevated parasite prevalence associated with living near the border suggests transmission resulting from border foci and residual confounding by environmental conditions that were mis-specified. Low spatial resolution temperature and rainfall data were used, a major limitation in an area with considerable ecological variation that drives local transmission dynamics. Similarly, low temporal resolution data were used for NDVI, which was estimated biannually. Further, this analysis assumed linear relationships between elevation, NDVI, temperature, rainfall, and parasite prevalence, presumably oversimplifications of their true associations. To the extent that environmental factors confer risk in a concerted way and that such risk is largely unmodifiable, precise decomposition may be both unnecessary and unactionable. However, the cumulative effect of environmental risk factors and unmeasured confounding, including that imparted by unmeasured human and vector movement, associated with distance from the border should be considered and has been estimated.^8^

Regional coordination for cross-border malaria reduction may improve programmatic impact and has been conducted in southern Africa, the Greater Mekong Subregion, and the Arabian Peninsula. The Lubombo Spatial Development Initiative among Mozambique, South Africa, and Eswatini, which included IRS expansion into Maputo Province, Mozambique, led to 78% and 96% declines in case counts in Mpumalanga and KwaZulu-Natal, South Africa, and a 95% decline in Eswatini after 4 years.^33^ Similarly, under the Trans-Kunene malaria initiative, Namibian villages bordering those in Angola with synchronized LLIN distribution and monthly behavior change programming saw a 17% decrease in fever among children under age 5 years.^32^ In Yunnan Province, China, and neighboring areas of Myanmar, malaria care provider establishment, LLIN distributions, behavior change communications, and community case management targeting migrant and border communities coincided with a 95% reduction in the annual parasite index over 7 years in China and an 83% reduction over 5 years in Myanmar.^34^ Finally, in Yemen and Saudi Arabia, the rollout of coordinated border IRS, space spraying, active case detection, and case management at health posts coincided with a 90% decrease in incidence in Saudi Arabia, although the reduction may have been attributable to a change in first line treatment and decreased rainfall.^35^^,^^36^ These initiatives provide possible models for coordinated programming in contiguous districts of Zimbabwe and Mozambique.

Our study highlights the importance of malaria control aimed at mitigating local transmission along the Zimbabwe-Mozambique border and points to the need for future research to identify drivers of residual transmission. In addition, our study reaffirms the E8’s objectives of regional coordination, policy harmonization, and cross-border malaria reduction.^6^ Lastly, our study supports E8’s call for targeted messaging to residents of border areas on LLIN, repellant, and chemoprophylaxis use during travel when malaria risk is elevated.^5^
